# Ovarian stimulated cycle: not a better alternative for women without ovulation disorder in intrauterine insemination

**DOI:** 10.18632/oncotarget.22052

**Published:** 2017-10-23

**Authors:** Kemei Zhang, Yinjiao Shi, Ensheng Wang, Li Wang, Qingbo Hu, Yibo Dai, Haiyan Xu, Jiaou Zhang, Ping Jin, Xueqin Chen, Jing Shu

**Affiliations:** ^1^ Reproductive Medicine Center, Ningbo First Hospital, Zhejiang 315010, China

**Keywords:** clinical pregnancy rate, intrauterine insemination (IUI), ovarian stimulated cycle, natural cycle, non-ovulation disorder

## Abstract

To explore the related factors on the clinical pregnancy outcome in intrauterine insemination, a retrospective study was conducted on the clinical data of 580 cycles for 301 infertile couples who were treated with intrauterine insemination. The female age, male age, duration of infertility, treatment protocols, endometrial thickness and sperm parameters were compared between pregnant group and non-pregnant group. The results showed that there were statistical differences in female age, duration of infertility and endometrial thickness between the two groups. The pregnancy rate was 19.34% in Group A (female age ≤ 30 y) compared with 10.91% in Group B (female age > 30 y). The pregnancy rate was 18.44% when the duration of infertility ≤ 2 years, which was higher than another group 10.73% when the duration of infertility > 2 years. Group analysis according to endometrial thickness (Group1: < 8 mm; Group 2: ≥ 8 mm and ≤ 12 mm; Group 3: > 12 mm) demonstrated significant differences in clinical pregnancy rate (7.41%, 18.00% and 11.48% respectively). For those infertile female without ovulation failure, the higher clinical pregnancy rates were observed in patients undergoing intrauterine insemination in natural cycle 16.12% when compared with the patients in ovarian stimulated cycles 10.48%. Thus, we demonstrate that the pregnancy rate is related with female age, duration of infertility and endometrial thickness. The ovarian stimulated cycle couldn’t improve the pregnancy outcome for those women without ovulation disorder in intrauterine insemination.

## INTRODUCTION

Intrauterine insemination (IUI) is the first-line approach for infertile couples in the assisted reproductive treatment (ART) procedures, which is used widely for a broad range of indications in the reproductive medicine. It is commonly applied to the infertile couples diagnosed with the mild male factor, endometriosis, ovulation failure and unexplained factors [1]. Treatment with IUI is simple, less invasive and less expensive, with a lower multiple delivery rate and lower complication than *in vitro* fertilization (IVF)/ intracytoplasmic sperm injection (ICSI) [2, 3]. Therefore, IUI technology is widely used around the world. The data from the European Society of Human Reproduction and Embryology (ESHRE) showed that 162,843 IUI cycles were performed in 2009 compared with 135,621 IVF cycles during the same period [2]. However, the pregnancy rate per cycle with IUI is otherwise compared with IVF/ICSI [2, 4]. The sperm preparation techniques have been improved much in recent decades [5, 6], however, the clinical pregnancy rate with IUI remains unchanged [7, 8]. According to the reports from a large number of reproductive centers, the clinical pregnancy rate with IUI per cycle is between 11.4% and 12.6% [1]. Thus, how to improve the outcomes of treatment with IUI still remains elusive for all reproductive doctors. Here, data from 580 cycles for 301 infertile couples in our center was analyzed to define which the following factors, including female age, male age, duration of infertility, treatment protocol, endometrial thickness, sperm parameters and treatment cycles, contribute to the positive clinical pregnancy outcomes.

## RESULTS

A total of 580 IUI cycles from 301 couples were enrolled in the study from January 1, 2015 to February 28, 2017, including 392 cycles in primary infertility and 188 cycles in secondary infertility. According to the statistics, 121 couples performed one IUI cycle, and 109 couples as well as 40 couples performed two and three cycles. Only 31 couples performed more than four IUI cycles. The mean age of females was 30.3 years old (varied from 20 to 45), and it was 31.6 years old in males (varied from 23 to 46). The mean infertility duration was 2.57 years (varied from 1 to 10). There were 89 cases diagnosed as clinical pregnancy. The clinical pregnancy rate was 15.34% (89/580), the abortion rate was 16.85% (15/89), the twin pregnancy rate was 6.52% (3/89), and the ectopic pregnancy rate was 4.49% (4/89).

As presented in Table 1, the infertile females were significantly younger in pregnant group (29.36 ± 3.16) than those in non-pregnant group (30.45 ± 3.68), the difference of which was statistical (*p <* 0.01). There was no difference in the age of males between pregnant group (31.24 ± 4.09) and non-pregnant group (31.67 ± 4.10) (*p >* 0.05). The clinical pregnancy rate was 19.34% (59/305) in females whose ages were below 30 years old, and that was 10.91% (30/275) in those females who were above 30 (*p <* 0.01), as shown in Table 2. The clinical pregnancy rate had no significant difference between the males whose ages were below 30 years old and those whose ages were above 30 (18.03% to 13.39%, *p >* 0.05).

**Table 1:** Comparison of the female age, male age, duration of infertility, endometrial thickness on hCG day and PR between pregnant group and non-pregnant group

	Female age (y)	Male age (y)	Duration of infertility (y)	Endometrial thickness (mm)	PR (%)
Pregnant group	29.36 ± 3.16	31.24 ± 4.09	2.12 ± 1.32	9.94 ± 1.86	31.72 ± 9.27
Non-pregnant group	30.45 ± 3.68	31.67 ± 4.10	2.65 ± 1.60	9.41 ± 2.17	32.32 ± 9.68
*P* value	0.009^**^	0.362	0.003^**^	0.032^*^	0.592

**Table 2:** The clinical pregnancy rate according to female age, male age and duration of infertility

	Female age (y)			Male age (y)		Duration of infertility (y)			
	≤ 30	> 30	≤ 30		> 30		≤ 2		> 2
Pregnant group	59	30	44		45		64		25
Non-pregnant group	246	245	200		291		283		208
Clinical pregnancy rate(%)	19.34%	10.91%	18.03%		13.39%		18.44%		10.73%
χ^2^ value	7.921			2.343				6.386	
*P* value	0.005^**^			0.126				0.012^*^	

The duration of infertility was significantly different between the pregnant group and the non-pregnant group (2.12 ± 1.32 years to 2.65 ± 1.60 years, *p <* 0.01), as presented in Table 1. The clinical pregnancy rate was 18.44% (64/347) in the group whose infertility duration was less than 2 years, and was 10.73% (25/233) in those whose duration were more than 2 years (*p <* 0.05), as presented in Table 2.

Our results showed that women with normal menstruation were performed 452 cycles, including 242 natural cycles and 210 ovarian stimulated cycles. The clinical pregnancy rate was higher in natural cycles than in ovarian stimulated cycles (16.12% to 10.48%). However, the difference was not statistical (*p >* 0.05). As shown in Table 3, other women with ovulation dysfunction were performed 210 ovarian stimulated cycles. The clinical pregnancy rate was 13.33% in CC/CC+Gn group, 23.26% in letrozole/ letrozole+Gn group, and 33.33% in Gn group (*p >* 0.05).

**Table 3:** The clinical pregnancy rate according to the treatment protocols

		Natural cycles		Ovrian stimulated cycles		χ^2^ value	*P* value
Non-ovulation disorder	Pregnant group	39		22		3.063	0.080
	Non-pregnant group	203		188			
	Clinical pregnancy rate (%)	16.12%		10.48%			
Ovulation disorder		CC/CC+Gn	Latrozole/latrozole+Gn		Gn		
	Pregnant group	4	20		4		
	Non-pregnant group	26	66		8	2.299	0.317
	Clinical pregnancy rate (%)	13.33%	23.26%		33.33%		

The endometrial thickness on hCG day was 9.94 ± 1.86mm in pregnant group and 9.41 ± 2.17mm in non-pregnant group (*p <* 0.05). As shown in Table 4, the cases were divided into three groups according to the membrane on the day of hCG trigger (Group1: < 8 mm; Group 2: 8–12 mm; Group 3: > 12 mm). The pregnancy rates were statistically different among the three groups (7.41%, 18.00%, 11.48%, *p <* 0.05).

**Table 4:** The clinical pregnancy rate according to the endometrial thickness on hCG day

	< 8 mm	8–12 mm	> 12 mm	χ^2^ value	*P* value
Pregnant group	8	74	7	8.180	0.017^*^
Non-pregnant group	100	337	54		
Clinical pregnancy rate (%)	7.41%	18.00%	11.48%		

The progressive motility of males’ sperm was 31.72 ± 9.27% in the pregnant group and 32.32 ± 9.68% in the non-pregnant group (*p >* 0.05). As presented in Table 5, the cases were divided into three groups according to the progressive motility of males’ sperm (Group 1: ≤ 20%; Group 2: 20–32%; Group3: ≥ 32%), and the pregnancy rates were not significantly different (11.94%、16.39%、15.27%, *p >* 0.05).

**Table 5:** The clinical pregnancy rate according to the PR before washing

	≤ 20 %	20–32%	≥ 32%	χ^2^ value	*P* value
Pregnant group	8	39	42	0.798	0.671
Non-pregnant group	59	199	233		
Clinical pregnancy rate (%)	11.94%	16.39%	15.27%		

**Figure 1:**
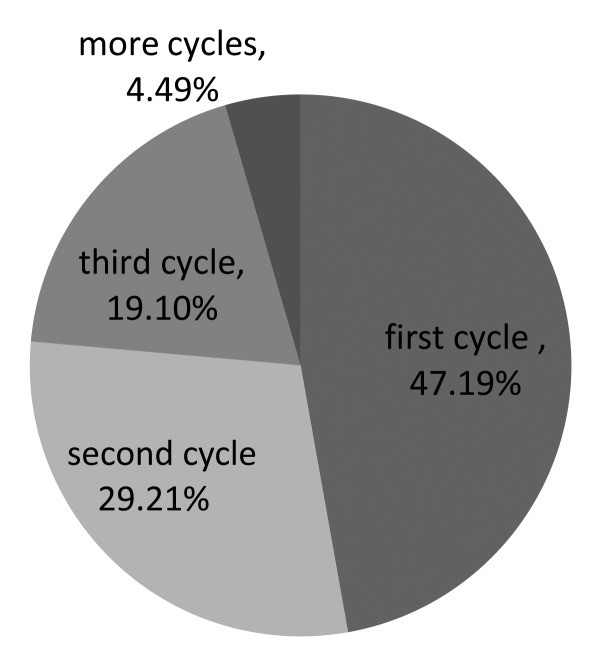
The clinical pregnancy rate according to the cycles in pregnant patients

As presented in Table 6, the cases were divided into four groups according to the times of IUI cycles (first cycle, second cycle, third cycle and ≥ forth cycle), and the pregnancy rates were not significantly different (14.09%, 14.44%, 23.94%, 12.90%, *p >* 0.05). And the accumulated pregnancy rates were 14.09%、30.63%、61.15%, 76.72%, respectively. Among the pregnant group (89 women got pregnant totally), 47.19% (42/89) patients got pregnant in the first time, and 29.21% (26/89) patients as well as 19.10% (17/89) patients got pregnant in the second and third time, respectively. Only 4.49% (4/89) patients got pregnant four times later, as shown in Figure 1.

**Table 6:** The clinical pregnancy rate according to the operation times

	First cycle	Second cycle	Third cycle	≥ Fourth cycle	χ^2^ value	*P* value
Pregnant group	42	26	17	4	4.655	0.199
Non-pregnant group	256	154	54	27		
Clinical pregnancy rate (%)	14.09%	14.44%	23.94%	12.9%		

	One cycle	Two cycles	Three cycles	≥ four cycles
Pregnant couples	42	68	85	89
Infertile couples	298	222	139	116
Accumulative pregnancy rate (%)	14.09%	30.63%	61.15%	76.72%

## DISCUSSION

The number of infertile couples has increased a lot in recent years due to the environmental pollution and work pressure. Thanks to the rapid development of ART, more and more infertile couples choose this technique to resolve their reproductive problems. IUI is the first-line treatment during ART procedure [9], which makes sperm bypass the cervical-barrier to increase the number of sperm surrounding the egg. The sperm are washed to remove the inactive sperm, immature germ cells, microorganisms, white cells and antigen proteins. By this way, the activity and quality of sperm are meliorated enormously. IUI is a simpler, safer and cheaper treatment compared with IVF/ICSI. However, the success rate of IUI is lower than other ART, which limits the application of this technology. In this study, we find out some factors which contribute to the outcome of IUI treatment.

### Age

In the present research, we found that the female age was different between pregnant group and non-pregnant group. The women were younger in pregnant group than those in non-pregnant group, in agreement with the literatures [7, 10–13]. The differences were statistically significant. When the woman was older, the clinical pregnancy rate with IUI was lower accordingly. Otherwise, there was no difference in the effect of male age on the pregnancy rate. As we know, female age is an independent factor affecting pregnancy outcome [7, 14]. When the women become older, especially over 35 years old, the amount of oocyte exhausts rapidly. The accumulation of metabolites in the body changes the ovarian environment, such as the mutations in DNA and shortening of telomeres, which physiologically declines the quality of oocytes, increasing the chance of infertility [15–20]. It has been reported that the optimal reproductive age for women is 20 to 30 years old, and the ability of reproduction declines rapidly after 30 years old [7, 14]. In modern society, especially in some developed cities, many couples delay the timing of having children, which brings a lot of reproductive problems. The spontaneous abortion rate and fetal malformation rate go up accompanied by the maternal age. It has been shown that the pregnancy rate prominently reduces when the age of women is over 37 years old and is very little when over 40 years old. In our report, there was no effect in the male age on the pregnancy rate. Therefore, we should consider the female age as an important factor while determining the therapeutic regimen.

### Duration of infertility

In our study, we found that the duration of infertility was different between pregnant group and non-pregnant group. It was significantly shorter in pregnant group than in non-pregnant group. Furthermore, the clinical pregnancy rate per cycle of couples was higher when their duration of infertility was below 2 years, compared with couples over 2 years. This report is consistent with previously reported [11]. Thus we come to conclusion that the duration of infertility is highly associated with the pregnancy rate. The pregnancy rate per cycle with IUI would decrease when the duration of infertility is longer.

### The endometrial thickness on hCG day

According to the statistics, the average endometrial thickness was different between pregnant group and non-pregnant group. When the patients were divided into three groups according to the membrane on the day of hCG trigger (Group1: < 8 mm; Group 2: 8–12 mm; Group 3: > 12 mm), we found that the pregnancy rate was highest in the group when the endometrial thickness ranged from 8 to 12 mm compared with other two groups, which was consistent with the previous studies [21, 22]. Weissman et al. [23] reported that the pregnancy rate is proportional to the endometrial thickness. However, the pregnancy rate and planted rate decline when the endometrial thickness is over 14mm. It will lead to implantation failure when the endometrial thickness is too thick or too thin [23, 24]. Thin endometrium was mostly caused by the low estrogen, endometrial damage or inflammation. Low estrogen usually dues to the poor oocyte quality and poor ovarian function, which may decline the pregnancy rate by affecting the quality of embryo and endometrial receptivity. On the other hand, the implantation is affected by the endometrial damage or inflammation in another way. The decline of the pregnancy rate might be related to the endometrial damage caused by the transfer tube with IUI when the endometrial thickness is too thick.

### Treatment protocol: natural cycle and ovarian stimulated cycle

In current study, we found that there was no statically difference in the clinical pregnancy rate per cycle with IUI between natural cycle and ovarian stimulated cycle for those women with regular menstruations and normal ovulation. It has been revealed that ovarian stimulated treatment could not improve the outcome of the IUI for the infertile women with non-ovulation disorder. At the same time, there was no difference in the pregnancy rate among groups with different ovarian stimulated protocols. It was announced that the pregnancy rate with ovarian stimulated cycles is higher than that with natural cycles [25, 26]. In our opinion, the opposite conclusion is due to the different cohort chosen in the different research. In the previous study, people with or without ovulation dysfunction were combined for the statistics. The ovarian stimulation treatment would be efficient obviously for those women with the ovulation dysfunction, whom the natural cycle could not be used for. Therefore, it is not difficult to come to a false conclusion that ovarian stimulated cycle is better than natural cycle.

As shown in Table 3, the clinical pregnancy rate in natural cycle group is higher than in ovarian stimulated cycle group (16.12% to 10.48%), although without statistics difference (Table 3). In the clinic, we found that the infertile couples with mild male factors, such as sexual dysfunction, preferred to choose natural cycle in IUI treatment. Those infertile patients were easy to get pregnant in IUI cycle. In our opinion, that’s the reason why the outcome in natural cycle group was better than in ovarian stimulated cycle group.

In general, the ovarian stimulated cycle is not a better alternative compared with the natural cycle for the women without ovulation disorder in IUI.

### Sperm parameter

In our research, there was no difference in PR before sperm was washed between the pregnant group and non-pregnant group. The pregnancy rate was highest in the group when the PR was from 20% to 32%. Stone et al. [14] also reported that sperm motility in inseminate was a major determinant of outcome, with PR < 20% significantly decreasing the pregnancy outcome. As we know, the IUI treatment is a technology through putting sperm into the uterine to increase the amount of sperm around the egg. Thus, male patients with mild asthenospermia are the best subjects who receive this treatment. However, when the activity of sperm declines further, the amount of sperm can hardly reach the enough concentration to fertilize the egg. On the other hand, the female can get pregnant without IUI if the sperm are normal. In this point, there must be some other factors which affect the pregnancy outcome for those infertile couples, such as sexual dysfunction, fallopian tube inflammation, endometriosis and unexplained reasons. It is very difficult to get pregnant with IUI for people with those reasons mentioned above, except sexual dysfunction. That is why the pregnancy rate was lower with IUI in the group when the sperm was normal compared with the group when the sperm was abnormal mildly. We can see the tendency even if there was no significant difference among three groups. We will expand the sample size to confirm this regularity in our future investigation.

### Treatment cycles

Most of the infertile couples performed 1–3 cycles with IUI treatment in our reproductive center. Seven couples performed 5cycles, and two couples performed 6 cycles. It is suggested that the infertile couples with unexplained reasons should be treated with IUI for at least 3 times before turning to further assisted reproductive technique, such as IVF/ICSI.

Our data showed that the cumulative pregnancy rate increased gradually with the increase of cycles. The cumulative pregnancy rate was up to 61.15% when the couples preformed 3 cycles with IUI. According to the analysis in the pregnant group, 47.19% patients got pregnant in the first time, and 29.21% patients as well as 19.10% patients got pregnant in the second and third time, respectively. Only 4.49% patients got pregnant four times later. Although the cumulative pregnancy rate increases gradually by the increase of cycles, the chances of getting pregnant per cycle diminishes conversely, which is similar with Dinelli et al. [7]. We thought that the pregnancy rate was related to the causes of infertile couples. Some patients with mild female factor, cervical factor and mild endometriosis can get pregnant with IUI in the first three times. There are still some potential factors which are hardly to be discovered by the current technology. It is difficult to get pregnant with IUI by increasing the cycles for these patients.

Collectively, the IUI treatment is a kind of simple, cheap and invasive technique compared with IVF/ICSI. There is lower success rate but less intervention in the reproductive process with IUI compared with other assisted reproductive techniques. We suggest those infertile couples who may get pregnant with IUI to perform this first-line technology in reproductive treatments. More importantly, natural cycle may get a better outcome for those people with non-ovulation dysfunction in the IUI treatment. In other words, the ovarian stimulated cycle is not a better alternative for infertile women with normal ovulation.

## MATERIALS AND METHODS

### Study design

A retrospective study was performed by reviewing the clinical data of 580 IUI cycles from 301 couples at the Reproductive Center of Ningbo First Hospital in China during January 2015 and February 2017. The inclusion criteria for IUI included: infertility for at least one year, at least one patent fallopian tube. Duration of infertile: from the time since a couple have sex without any contraception. Informed consent was obtained from all subjects. The study protocol was approved by the Institutional Review Board of Ningbo First Hospital, China. All methods were performed in accordance with the approved guidelines.

### Treatment protocols

#### Natural cycle

Natural cycle was administered in females whose menstrual cycles were regular. Intrauterine insemination was administered according to the peak of the luteinizing hormone (LH) which was measured day by day since the diameter of follicle got to 16–18mm.

Ovulation induction was performed as the following protocol:

Clomiphene citrate ( CC) 50–100 mg/day starting from day 3–5 for 5 days.

Letrozole (LE) 2.5–5.0 mg/day from day 3–5 for 5 days.

HMG 37.5–75 IU/day starting from day 3–5 for a variable duration depending on the response.

CC combined with HMG – CC 50–100 mg/day starting from days 3–5 for 5 days followed by the addition of 37.5–75 IU of HMG for a variable duration depending on the response.

LE combined with HMG – LE 2.5–5.0 mg/day starting from day 3–5 for 5 days followed by the addition of 37.5–75 IU of HMG for a variable duration depending on the response.

Intrauterine insemination (IUI) was administered according to the peak of the luteinizing hormone. And the cycle would be canceled to avoid the ovarian hyperstimulation syndrome (OHSS) if the estradiol (E2) was higher than 1500 pg/ml or the mature follicles were more than 3.

### Operative time

When at least one mature follicle had a diameter of 18 mm or more and the endometrial thickness achieved 7 mm, we triggered ovulation with intramuscular injection of urinary human chorionic gonadotrophin (hCG) (5000–10 000 IU), or hypodermic injection of recombinant human chorionic gonadotropin alfa (Ovidrel, 0.25 mg), or hypodermic injection of Triptorelin (0.1 mg). Insemination was performed 36–40 hours after injection.

### Semen treatment

Semen was collected by masturbation after abstinence for 3–7 days and prepared with two-layer density gradient centrifugation after liquefaction. Mechanical method was used for abnormal semen liquefaction. The volume of washed semen sample used for insemination was 0.3–0.5 ml.

### Luteal phase support

The luteal phase was used routinely in all patients, starting from the day since IUI was performed. It consisted of Duphaston (Dydrogesterone Tablets, 20 mg/day, Abbott, Netherlands) for 14 days. A blood test for hCG assay was performed in 14 days after insemination to confirm whether pregnancy had occurred. In women with positive hCG, ultrasound examination was performed at 7 weeks’ gestation to confirm fetal viability. A clinical pregnancy was defined as one in which there was ultrasonographic evidence.

### Statistical analysis

The data expresses the means ± SD. The baseline differences between the two groups were analyzed by Student’s *t* test. Pearson’s Chi-square test was used to compare the ratios between groups. A value of p less than 0.05 was considered statistically significant. The data was analyzed using the Statistical Package for the Social Sciences (SPSS) for Windows (version 19.0).
